# Beyond additive genetic effects: Explaining family resemblance in school performance across millions of pairs of Norwegian relatives

**DOI:** 10.1073/pnas.2419627122

**Published:** 2025-06-20

**Authors:** Nikolai Haahjem Eftedal, Espen Moen Eilertsen, Hans Fredrik Sunde, Thomas Haarklau Kleppestø, Eivind Ystrom, Nikolai Olavi Czajkowski

**Affiliations:** ^a^Promenta Research Centre, Department of Psychology, University of Oslo, Oslo 0373, Norway; ^b^Centre for Fertility and Health, Norwegian Institute of Public Health, Oslo 0456, Norway; ^c^Department of Psychology, Norwegian University of Science and Technology, Trondheim 7491, Norway; ^d^PsychGen Centre for Genetic Epidemiology and Mental Health, Norwegian Institute of Public Health, Oslo 0456, Norway

**Keywords:** intergenerational transmission, education, assortative mating

## Abstract

We investigate the hypothesis that family resemblance on school performance can be fully explained by additive genetic effects and assortative mating. Our sample consists of all schoolchildren who took Norwegian national standardized tests between 2007 and 2019 (N = 936,708). These tests measure aptitude in math and reading comprehension, and are taken the years children turn 10, 13, and 14 y old. We identify millions of pairs of relatives within our sample (82 different kinds, in total), including not only conventional biological relatives such as siblings and cousins, but also relatives-in-law, relatives through adoption, twins, and relatives connected through twins. When fitting models which assume that family resemblance arises solely from additive genetic effects and assortative mating, we find that they describe much of our data well, but that they systematically underestimate the similarity of close relatives (particularly monozygotic twins), maternal relatives, relatives-in-law, and relatives through adoption. We discuss potential explanations for these deviations, including shared-environmental effects, nonadditive genetic effects, and gene–environment interplay.

## Significance

Understanding why educational outcomes run in families is important for both policy and research. Here, we report and analyze correlations between millions of pairs of relatives, of a wide range of categories, on psychometrically rigorous standardized tests, from a large sample with minimal sampling biases. Our findings provide challenges both to recent claims that additive genetic effects and assortative mating are sufficient to explain family resemblance, as well as to claims that genetic factors have only a very limited role. By publicly sharing our correlation data, we enable other researchers to fit and evaluate alternative models of their choosing, to further advance our understanding of why relatives resemble each other on socially relevant outcomes.

Relatives tend to resemble each other in their educational and occupational outcomes. This is well documented for close relatives (1, 2), and there is an increasing number of studies showing substantial correlations also between nonimmediate relatives (3–9).

There has long been controversy around the importance of genetic effects in explaining this family resemblance, with opinions ranging from the view that genes are the main reason relatives have similar socioeconomic outcomes, to the view that they play hardly any role. An important contribution to this debate was recently made by Clark (8), who studied an English genealogical database of 422,374 people going back to the 17th century. He reported correlations on socioeconomic outcomes for a wide range of relatives, going out as far as 4th cousins, on outcomes such as occupational status, higher education status, literacy, and dwelling value. To these correlations, Clark fitted models based on theory from Fisher (10), in which only genes can explain similarity between relatives, and found that they could explain his set of correlations almost perfectly, with values of R^2^ above 0.95. Importantly, these models, which we will refer to as “Fisherian,” account for the effects of assortative mating: the tendency for people to have children with others who are similar to themselves. With random mating, the expected genotypic similarity between relatives rapidly diminishes with increasing genealogical distance, going from 1/2 for siblings, to 1/8 for 1st cousins, and further on to 1/32 for 2nd cousins, meaning that they are about as genotypically similar as random pairings of people, and that they will be predicted to be phenotypically uncorrelated as well, no matter how high the heritability is. But, as Fisher showed, when there is assortative mating, these expected correlations of genetic propensities can start off higher for siblings and drop off less steeply. For distant relatives, they can then be many times higher than under random mating. And the phenotypic correlations reported by Clark were indeed so high that his models estimated genotypic mate correlations at 0.57 to be able to account for them.

Clark does not claim to have decisively proven that the family resemblance he observed is fully due to additive genetic effects. His conclusion is rather that “whatever social processes are producing the observed outcomes have a form of transmission which mimics additive genetic effects” (p. 1). Related to a similar debate decades earlier, about how best to model IQ-correlations between close relatives such as those gathered by Burt (11), Cloninger et al. (12) showed that, in most cases, it is possible to make models with cultural transmission that give the same predictions as Fisherian models. The converse of this does not hold, however: predictions from cultural models are not necessarily straightforward to produce in models with only genetic transmission. The fact that Clark’s fully genetic model with only two free parameters had excellent fit was then by no means a foregone conclusion (13). However, Collado et al. (3) argue that the correlations they report for a large sample of Swedish relatives are inconsistent with Fisherian models, particularly so for several categories of nonbiological relatives connected through marriages, which were not modeled by Clark. Collado et al., who studied outcomes similar to those in Clark, including educational attainment and income, then rather concluded that family resemblance is mostly due to cultural transmission. Notably though, Collado et al.’s models differ from Clark’s in that they restrict the genotypic mate correlation to equal the phenotypic mate correlation multiplied by the trait’s heritability. This has been argued to be part of the reason why the two papers reach such different conclusions (14).

We here seek to add to the discussion on how best to explain why relatives are similar, by presenting correlations between a wide range of relatives, from a large sample, on a set of theoretically relevant and psychometrically sound phenotypes. We hope others will use the correlations we report to fit and compare models of their choice. Here, as an initial investigation, we follow the modeling approach used by Clark, which assumes that family resemblance can be fully explained by additive genetic effects and assortative mating.

Our set of phenotypes, the Norwegian national standardized tests, are developed through rigorous procedures to measure “fundamental abilities in reading, math and English that are important for learning across all subjects” (15). The tests are then meant to measure aspects of school performance related to cognitive ability more so than aspects such as motivation and study habits, and they have indeed been shown to correlate strongly with measures of IQ (16). In addition to being interesting in their own right, our phenotypes are then relevant to the wider discussion around why socioeconomic outcomes run in families: Outcomes such as academic degrees or income cannot be written directly into one’s genome. Nor are they typically absorbed from the environment in any direct way. So, if these kinds of outcomes run in families, it seems likely that this is because more basic traits that causally lead to these outcomes also run in families. The abilities measured by the standardized tests could potentially be such causally mediating traits. Cognitive ability is among the strongest predictors we have for a wide range of relevant social outcomes (17), and there are indications that this in part reflects causal effects (18).

Our sample is everyone who took national tests between 2007 and 2019 (N = 936,708). As the tests are taken by more than 95% of children going through the Norwegian school system, our sample is largely free from sampling biases. And as our sample is large, and our registry data are extensive, we can identify millions of pairs of relatives, with most categories having at least several thousand pairs. The importance of having large samples, and thereby more precise correlation estimates, increases when studying distant relatives, since the theoretical impact of errors then tends to be larger. When studying close relatives, estimates of heritability will remain quite accurate even if the actual correlation should be 0.27 rather than 0.30, for example. But when studying distant relatives, correlations are generally lower, so an error of that same magnitude, e.g., going from 0.02 to 0.05, could mean that the implied heritability is more than doubled.

**Fig. 1.**
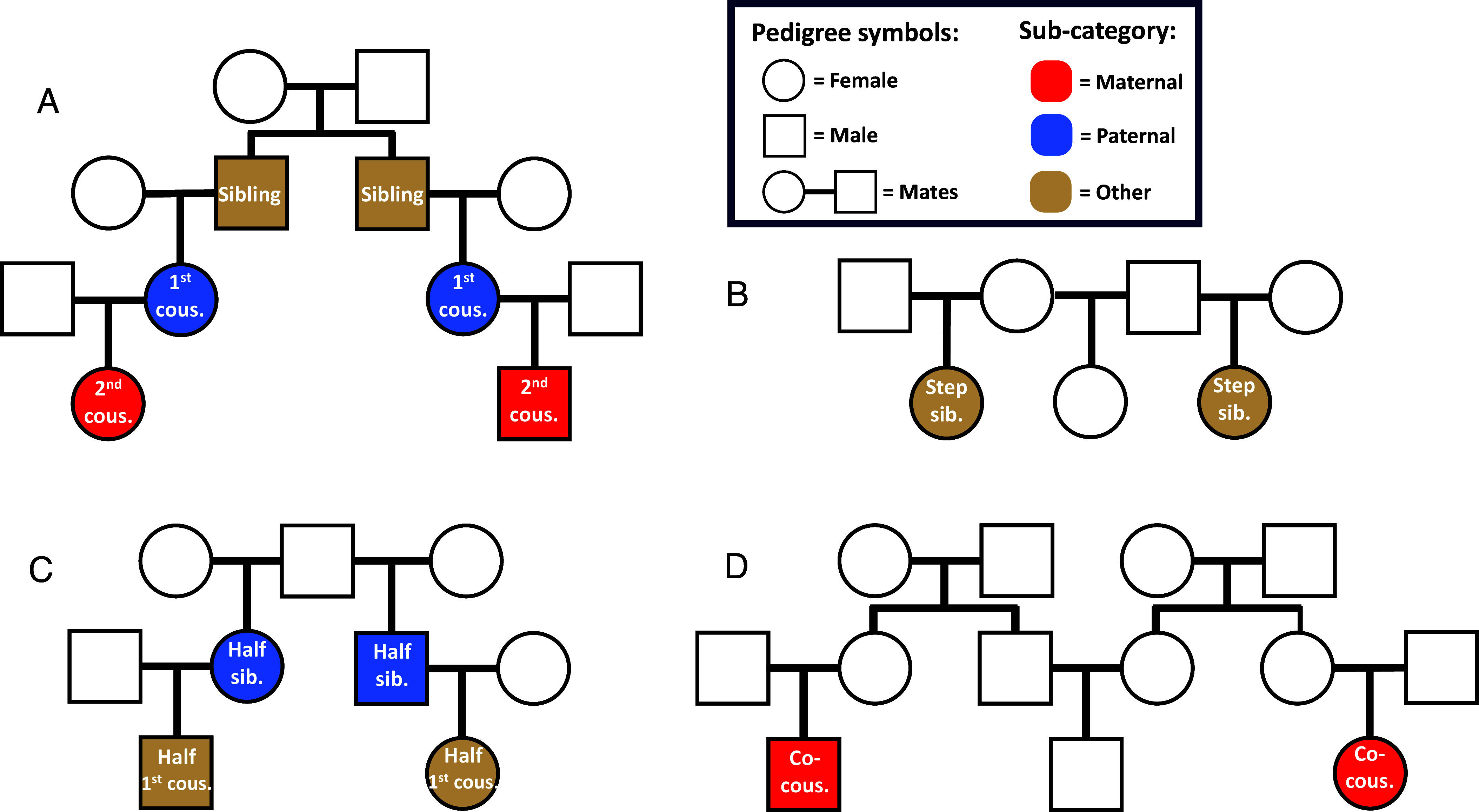
Illustrations of categories of relatives. Panel (*A*) shows a pair of siblings, in gold, a pair of 1st cousins, in blue, and a pair of 2nd cousins, in red. Panel (*B*) shows a pair of step siblings, in gold. Step siblings are relatives-in-law: they are not biologically related, but instead connected via mates. In their case, the mother of one of them had a child with the father of the other one. Panel (*C*) shows half siblings, in blue, and half 1st cousins, in gold. Note that the top part of *C* differs from the top of *A*, in that it shows one shared parent having two different mates, rather than just a single pair of mates as in *A*. Panel (*D*) shows Co-cousins, another category of relative-in-law. The mates connecting co-cousins are the uncle of one of them and the aunt of the other one. The colors in the figure signify the subcategorization of relatives as maternal, paternal, or other. The 2nd cousins in *A* are maternal 2nd cousins, because the pair of 1st cousins connecting them are both female. These female 1st cousins are paternal, because the siblings connecting them are brothers. These brothers cannot be categorized as either maternal or paternal, because they are connected through both their father and their mother. Similarly, the step siblings in *B* are neither maternal nor paternal, because they are connected via opposite sex mates. The half siblings in *C* are paternal half siblings, because they have the same father and different mothers. If they had instead been connected via having the same mother, they would have been maternal half siblings. The co-cousins in *D* are also maternal: for both co-cousins in this pair, it is the case that the mates connecting them are on their mother’s side of the family.

The categories of relatives we report correlations for here (Fig. 1) include monozygotic twins, dizygotic twins, siblings, half-siblings, 1st cousins, half 1st cousins, and 2nd cousins. We also have two categories of relatives-in-law, who are nonbiological relatives connected through marriages (with “marriage” here defined as a partnership with children, not necessarily legally married). These are step siblings and “co-cousins.” Step siblings are pairs who are connected through a marriage of one from each of their sets of parents. Co-cousin is a term we have made for relatives-in-law who are connected through having one or more cousins in common (i.e. a marriage between the uncle of one and the aunt of the other). A pair of co-cousins is then what you get when taking a 1st cousin from your father’s side of the family and then a 1st cousin from your mother’s side: this pair would then, in most cases, be unrelated if not for the connection between your mother and father. Beyond this, we have four categories of relatives through adoption: pairs of siblings, 1st cousins, 2nd cousins, and co-cousins, where at least one is an international adoptee. Within all these categories of relatives now mentioned, we make the following subdivisions, whenever applicable: 1) whether pairs are related through same-sex twins, 2) the genders of the parent(s) a pair is related through, and 3) the gender composition of the pair itself.

In total, this gives 82 correlations for each of the eight different tests. A unique aspect of this study lies in the simultaneous reporting of all these relations for the same set of phenotypes from the same sample. While numerous models can explain each individual correlation, the range of models capable of adequately accounting for all correlations simultaneously is narrower. When only looking at “standard” biological relatives (siblings, half-siblings, cousins, half cousins, etc.), a concern is that genetic and environmental sources of family resemblance could rise and fall in intensity at similar rates when moving from one type of relative to another, thereby making it hard for models to tell them apart. This kind of symmetry becomes less likely to hold when also including relatives-in-law and adoptive relatives, who could plausibly be closer environmentally than they are genotypically (*SI Appendix*, Fig. S1 and Table S8). Monozygotic twins, and their descendants, could also serve to break up this symmetry, through being more genotypically similar than their environmental distance would otherwise imply. The less it is the case that genetic and environmental effects mimic each other, the less able the Fisherian models will be to use genetic effects as substitutes for environmental effects. The extent to which this then leads to diminished performance from these models depends on how strong the environmental effects on family resemblance truly are. We thereby give the fully genetic Fisherian models new chances to fail and new chances to impress by not failing.

## Results

Our findings are broadly similar across all the eight different national tests. We here focus on the average score on the three tests given in the 8th grade (in math, Norwegian, and English). Averaging across tests minimizes error in measuring aspects common to all tests (within-person correlations between tests are high; see *SI Appendix*, Table S1). It also minimizes gender differences: the slight female advantage in reading balances a slight male advantage in math.

**Fig. 2.**
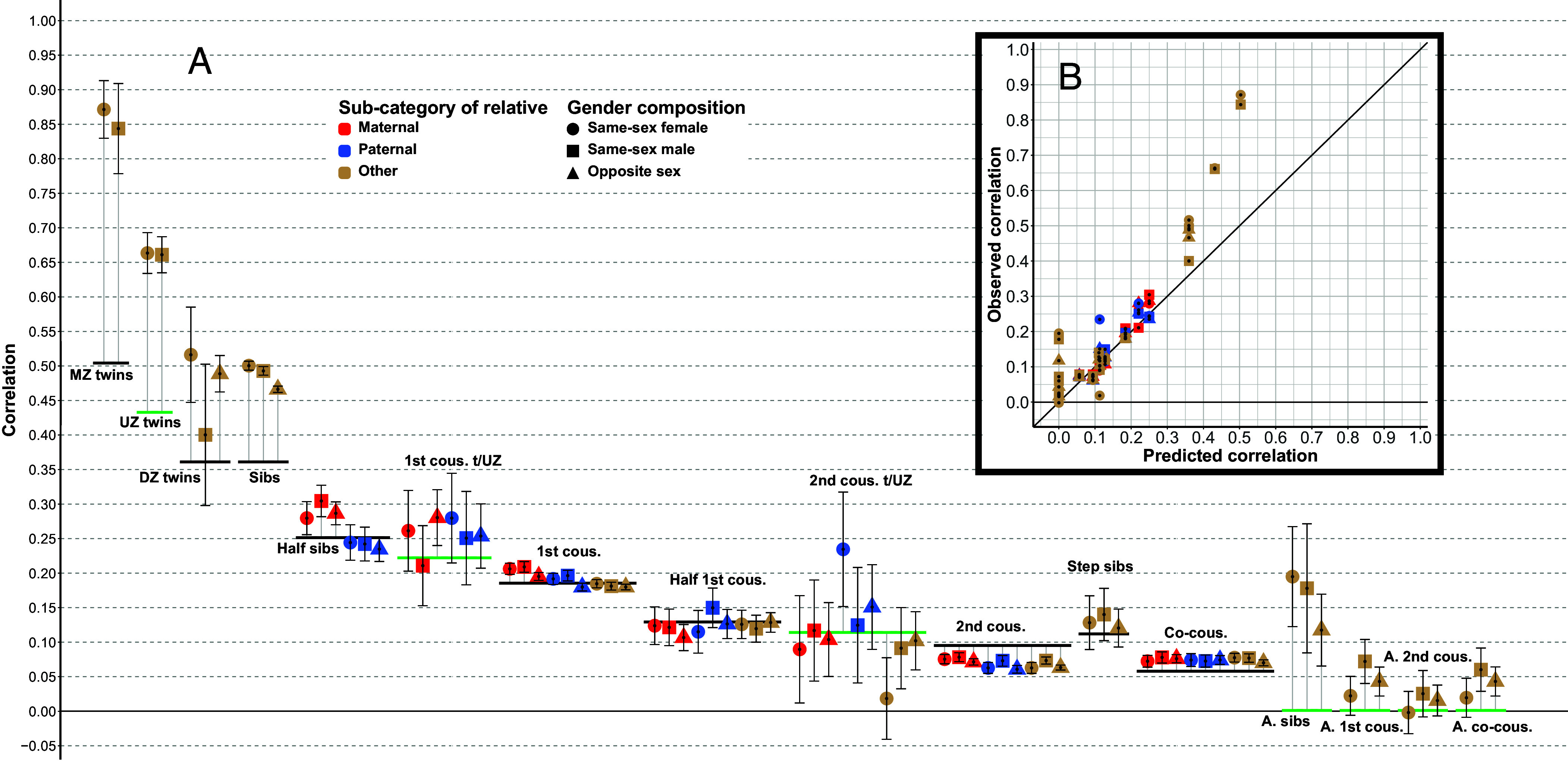
Correlations, and predictions from a model with *h*^2^ = 0.503, *m* = 0.430, and *c* = 0.972. For both *A* and *B*, the shapes and colors on the points indicate which subcategory of relative they belong to, and the gender composition of the pair, as explained in the figure legend. (*A*) Black horizontal bars are model predictions for correlations that were part of the model estimation. Green bars are predictions for categories of relatives that were not part of the model estimation. Predictions for categories of relatives involving twins of unknown zygosity are made on the assumption that the proportion of monozygotes among same-sex twins is 0.50. The points are observed correlations. Black lines stretching vertically from the points are 95% CI. Gray lines connect points to their predicted values. “MZ” is for “monozygotic,” “UZ” is for “unknown zygosity”; “DZ” is for “dizygotic”; “sibs” is for “siblings”; “cous.” is for “cousins”; “t/UZ” is for “related through twins of unknown zygosity”; “A.” is for “adoptive.” (*B*) Observed correlations are plotted against predictions, with a black diagonal line indicating where observations and predictions coincide.

Table 1 contains correlations for these averaged 8th grade test scores, for all our categories of relatives, subdivided according to whether pairs are related through their mothers, their fathers, or through opposite sex relatives. In Fig. 2, we further divide correlations according to the gender composition of the pair. Among the supplementary materials are files containing all these correlations for all the separate tests, as well as for averages of tests given in each of 5th, 8th, and 9th grade, and R scripts for running our analyses (19; see *SI Appendix*, section 1, for more information).

**Table 1.** Correlations, parameter estimates, and model predictions

		Bio + In-law			Bio only					
		*h*^2^ = 0.503 [0.404, 0.572] *m* = 0.430 [0.365, 0.478] *c* = 0.972 [0.864, 1.127] R^2^ = 0.873			*h*^2^ = 0.801 [0.749, 0.856] *m* = 0.225 [0.204, 0.246] *c* = 1.002 [0.896, 1.121] R^2^ = 0.988					
Relation	r [95% CI]	r̂	Δ	Δ%	r̂	Δ	Δ%	n	N pairs	N%
MZ twin	0.863 [0.844, 0.881]	0.503	0.360	71.6	0.801	0.062	7.7	0	795	0.02
*UZ twin*	0.662 [0.647, 0.676]	0.431	0.231	53.6	0.645	0.017	5.4	0|1	5,648	0.17
DZ twin	0.487 [0.466, 0.508]	0.360	0.127	35.3	0.490	0.003	0.6	1	5,116	0.15
Sibling	0.481 [0.478, 0.484]	0.360	0.121	33.6	0.490	0.009	1.8	1	285,025	8.33
Half sibling—mat	0.290 [0.279, 0.302]	0.250	0.040	16.0	0.301	0.011	3.7	2	25,891	0.76
Half sibling—pat	0.239 [0.227, 0.251]	0.250	0.011	4.4	0.301	0.062	20.6	2	22,942	0.67
*1st Cousin t/UZ*	0.258 [0.237, 0.279]	0.221	0.037	16.7	0.242	0.016	6.6	2|3	7,606	0.22
1st Cousin—mat	0.203 [0.199, 0.206]	0.184	0.019	10.3	0.184	0.019	10.3	3	240,187	7.02
1st Cousin—pat	0.188 [0.184, 0.192]	0.184	0.004	2.2	0.184	0.004	2.2	3	237,489	6.94
1st Cousin—opp	0.182 [0.179, 0.184]	0.184	0.002	1.1	0.184	0.002	1.1	3	455,840	13.32
Half 1st cousin—mat	0.115 [0.102, 0.128]	0.128	0.013	10.2	0.113	0.002	1.8	4	21,132	0.62
Half 1st cousin—pat	0.131 [0.116, 0.145]	0.128	0.003	2.3	0.113	0.018	15.9	4	17,048	0.50
Half 1st cousin—opp	0.126 [0.116, 0.136]	0.128	0.002	1.6	0.113	0.013	11.5	4	37,695	1.10
*2nd cousin t/UZ*	0.107 [0.087, 0.127]	0.113	0.006	5.3	0.091	0.016	17.6	4|5	9,100	0.27
2nd cousin—mat	0.075 [0.071, 0.078]	0.094	0.019	20.2	0.069	0.006	8.7	5	352,298	10.29
2nd cousin—pat	0.066 [0.062, 0.070]	0.094	0.028	29.8	0.069	0.003	4.3	5	249,651	7.29
2nd cousin—opp	0.068 [0.065, 0.070]	0.094	0.026	27.7	0.069	0.001	1.4	5	577,694	16.88
Step sibling	0.127 [0.108, 0.147]	0.111	0.016	14.4	0.067	0.060	89.6	2	10,167	0.30
Co-cousin—mat	0.076 [0.072, 0.080]	0.057	0.019	33.3	0.025	0.051	204.0	4	221,595	6.47
Co-cousin—pat	0.074 [0.069, 0.079]	0.057	0.017	29.8	0.025	0.049	196.0	4	186,182	5.44
Co-cousin—opp	0.074 [0.071, 0.077]	0.057	0.017	29.8	0.025	0.049	196.0	4	401,159	11.72
*Adopted sibling*	0.149 [0.111, 0.187]	0.000	0.149	∞	0.000	0.149	∞	—	2,536	0.07
*Adopted 1st cousin*	0.045 [0.030, 0.060]	0.000	0.045	∞	0.000	0.045	∞	—	17,361	0.51
*Adopted 2nd cousin*	0.014 [−0.002, 0.030]	0.000	0.014	∞	0.000	0.014	∞	—	15,230	0.44
*Adopted co-cousin*	0.042 [0.027, 0.056]	0.000	0.042	∞	0.000	0.042	∞	—	17,358	0.51

*Note.* Relation = Category of relative, with categories not part of model fitting highlighted in cursive. MZ = monozygotic; UZ = unknown zygosity; DZ = dizygotic; mat, pat, and opp = whether pairs are related through their mothers, fathers, or opposite sex pairs, respectively. r[95% CI] = correlations with 95% CI. Columns under the heading “Bio + In-law” show model predictions (r̂), and deviations from model predictions of the observed correlations, expressed as both raw values (Δ) and percentages (Δ%), for the model fitted to biological relatives, step siblings, and co-cousins, with dark blue numbers being positive and red numbers being negative. Predictions for categories of relatives involving twins of unknown zygosity are made on the assumption that the proportion of monozygotes among same-sex twins is 0.50. Estimated parameter values with 95% CI, and R^2^, for this model are shown just under the heading. *h*^2^ = heritability; *m* = genotypic partner correlation; *c* = the multiplier in the expression for half siblings and half 1st cousins. The columns under “Bio only” show this information for the model fitted to only biological relatives. n = degrees of relatedness, for biological relatives, or the combined distance to the connecting pair, for relatives-in-law; N pairs = the number of pairs of relatives in each category in our sample; N% = the percentage of our total number of pairs made up of pairs from each category.

When we calculate correlations between random pairings of unrelated people, we get estimates that do not significantly differ from zero in 95% of cases, suggesting that there are no aspects of our sample or our procedure which biases correlations to be nonzero.

The Fisherian models predict observed correlations from degrees of relatedness, and produce estimates of heritability, *h*^2^, and genotypic partner similarity, *m*. Correlations are weighted by their precision to account for differences in sample size across categories of relatives. Following Fisher’s formula, predicted correlations between 0th degree relatives (i.e., monozygotic twins) are simply equal to the trait’s heritability, and from there correlations drop by a constant rate, (1 + *m*)/2, as the distance in relatedness increases. This rate of decrease is lower the more genotypically assorted mates are, as indicated by *m.* Correlations between relatives-in-law and half-relatives can also be modeled, through adding multipliers to the formula, as explained in *Materials and Methods*. Adoptive relatives are not modeled, however, as we do not know their degree of relatedness. They are predicted to be uncorrelated in the Fisherian framework, since they are like random pairings of people, genetically speaking. We also do not model same-sex twins of unknown zygosity, nor descendants of such twins, again due to not knowing their exact degree of relatedness (*Materials and Methods*).

When fitting such a model to our correlations, the proportion of variance explained, R^2^, was 0.873. The estimated heritability, *h*^2^, was 0.503 and the estimated genotypic partner correlation, *m*, was 0.430. Table 1 contains predicted values for all correlations based on these estimates, as well as observed deviations from these predictions, expressed as both absolute values and percentages. The largest deviations from model predictions, whether measured in absolute terms or as percentages, were for monozygotic twins, who correlated substantially more strongly than predicted.

When excluding relatives-in-law from the model, R^2^ increases from 0.873 up to 0.988. Parameter estimates also change dramatically, with *h*^2^ increasing to 0.801, and *m* decreasing to 0.225. These values of *h*^2^ and *m* produce poor predictions for relatives-in-law: For step siblings, predicted correlations are about half as high as those we observe, and for co-cousins they are about a third as high (all predictions from this model are also included in Table 1). Excluding all twin correlations, on the other hand, only minimally affects parameter estimates and R^2^ (all changes <0.01), due to the small proportion of twin pairs in our sample (<1%).

For parameter estimates for all our phenotypes, from Fisherian models fitted to correlation sets both with and without relatives-in-law, as well as from applying the classical twin design (20) to only the correlations for monozygotic and dizygotic twins; see *SI Appendix*, section 2 and Table S2.

Half siblings and half 1st cousins have an additional multiplier in the formulas for their predicted correlations. Our estimated value of this multiplier did not differ significantly from 1, implying that the phenotypic partner correlation is not significantly different from the genotypic partner correlation, *m*. CI around this estimate are too wide to allow any strong conclusions, however.

Half siblings are also notable in that maternal pairs, who have the same mother, are substantially more similar than paternal pairs, who have the same father. Consistently, across all the eight separate national tests, the correlations between maternal half siblings are about 20% higher than those for paternal pairs. Maternal relatives are also significantly more strongly correlated among 1st cousins and 2nd cousins, but the magnitudes of these differences are smaller, whether expressed in absolute terms or as percentages. Also for co-cousins the maternal pairs are the most strongly correlated, but here the differences are not significant. The exceptions to this pattern of maternal correlations being larger are half 1st cousins, and also the categories of cousins related through same-sex twins of unknown zygosity (for whom sample sizes are comparatively small).

Regarding the gender compositions of the pairs of relatives themselves, a regression analysis, detailed in *Materials and Methods*, showed that correlations for same-sex pairs are systematically higher than those for opposite sex pairs, albeit slightly (b = 0.009, *P* < 0.001). The same kind of analysis comparing same-sex pair correlations for males versus females produced no significant difference (*P* = 0.45).

## Discussion

In a sample of 936,708 Norwegians, we report correlations on National test scores for a wide range of relatives. The national tests are psychometrically sound measures of “fundamental abilities in reading, math and English that are important for learning across all subjects” (15). We find that correlations are generally high relative to coefficients of genetic relatedness, increasingly so for relatives that are more distant. We investigated the hypothesis that this family resemblance can be fully explained by additive genetic effects and assortative mating, through fitting models based on the work of Fisher (10). Our conclusion is that these factors indeed appear to be important, but that a complete model would need other sources of family resemblance as well. Environmental effects appear necessary to fully account for correlations between adoptive relatives, between relatives-in-law, and between maternal relatives. Additionally, the high correlations we see between monozygotic twins are suggestive of nonadditive genetic effects and/or gene–environment interplay.

### Evidence for Environmental Sources of Family Resemblance.

Previous studies have shown that models attributing family resemblance entirely to genetic factors can accurately account for the correlations observed between biological relatives (3, 8). This is also the case in our data, where R^2^ is at 0.988 when the model is fitted to only our pairs of biological relatives (94% of which are either siblings, 1st cousins, or 2nd cousins). Consistent with Collado et al. (3), however, our models perform worse when nonbiological relatives are also included. R^2^ then drops to 0.873, even though we only add a total of twelve new correlations (step siblings and co-cousins, stratified by the genders of connecting relatives and of pairs themselves). The correlations between co-cousins are weighted heavily in the model estimation and calculation of R^2^, though, as they make up over 20% of our total amount of pairs.

Plausibly, genetic and environmental effects can “mimic” each other when looking only at biological relatives. Genetic effects are correlated most strongly between close relatives, and their correlation drops off at a constant rate as relatives become more distant: environmental effects could very well be correlated between relatives in a similar pattern, causing Fisherian models to interpret them as genetic.

Our correlations between adoptive relatives provide indications that environmental sources of family resemblance exist and that they indeed do mimic genetic effects in this way. Adoptive relatives will always be predicted to be uncorrelated in the Fisherian framework. The correlations between adoptive siblings, at ~0.15, are hard to explain without environmental effects. And the true shared-environmental variance component could also be larger than this, e.g., if there is selection on who chooses to, or is allowed to, adopt, so that the environmental variance for adoptees is reduced. Mirroring genotypic correlations between biological relatives, correlations between adoptive relatives decrease at a steady rate as they become more distant, dropping by ~70% when going from siblings to 1st cousins, from ~0.15 to ~0.04, and then by another ~70% when then going to 2nd cousins, down to ~0.01.

Potentially, the reason why Fisherian models perform less well when relatives-in-law are included could be that they break up this symmetry between genetic and shared-environmental effects. In a Fisherian model, with plausible values for *m*, co-cousins are about as genotypically similar as 3rd cousins are. But it could very well be that co-cousins are more strongly correlated on environmental effects than 3rd cousins. The same can be said comparing step siblings and 2nd cousins (*SI Appendix*, Fig. S1 and Table S8). An indication that co-cousins are environmentally closer than even 2nd cousins is our observed correlation between adoptive co-cousins, at 0.042, which is significantly higher than the one for adoptive 2nd cousins, at 0.014.

To the extent that environmental effects contribute to family resemblance, fully genetic models should then underestimate the similarity of relatives-in-law. This is also what we found: The estimates of *h^2^* and *m* that fit best with just the correlations between biological relatives (*h*^2^ = 0.801 and *m* = 0.225) produce severe underestimates of our correlations between relatives-in-law. The observed correlations between step siblings are ~90% higher than those predicted from these values, and the observed correlations between co-cousins are ~200% higher. The reduction in model fit when relatives-in-law are included, from R^2^ = 0.988 to R^2^ = 0.873, also aligns with the idea that the model now has a harder time using genetic effects to mimic environmental effects.

Shared-environmental effects could also explain that relatives related through their mothers are systematically more similar to each other in their test scores than corresponding relatives related through their fathers or through opposite sex pairs. Genetic similarity is not predicted to depend on whether relatives are maternal or paternal, but environmental similarity very well could depend on this. The difference is most pronounced for half siblings with the same mother, but it also appears for 1st cousins through sisters, and 2nd cousins through female 1st cousin pairs, making half 1st cousins through half sisters the only clear exception among our major categories of biological relatives. For co-cousins, who are not biological relatives, the estimated correlation is again highest for maternally related pairs, but the differences are not significant.

There are many ways to interpret elevated correlations between maternal relatives (21). They can represent the impacts of any traits of mothers, such as in-utero environments, parenting, nutrition, choice of where to live, and the choice of how much contact to have with one’s relatives. They can also reflect misattributed paternities in the registry, though the rate of this is likely to be below 2% in our sample (22). And they could reflect the influence of mitochondrial DNA, which is inherited fully along the maternal line. In the case of half siblings, it is relevant to consider that maternal half siblings are much more likely to grow up together in the same household than paternal half siblings are (23).

Last, environmental effects could account for our observation that same sex relatives tend to be slightly more similar than opposite sex relatives, which, again, is not predicted in the Fisherian framework. Fully genetic explanations of this pattern can still be made, however, such as gene-by-gene interactions involving genes on the sex chromosomes (24).

### Evidence for Genetic Sources of Family Resemblance.

While we find clear indications of environmental sources of family resemblance, we consider it likely that genetic effects also contribute. Otherwise, it is hard to explain that adoptive siblings are so much less similar to each other than biological siblings are, with correlations at ~0.15 compared to ~0.50 for biological siblings. Similarly, biological 1st cousins and 2nd cousins are several times more strongly correlated than the corresponding adoptive relatives. This speaks against recent suggestions that the heritability of educational outcomes might be as low as 7% (3).

The fact that correlations between monozygotic twins (“rMZ”) are much higher than those between dizygotic twins (“rDZ”) and regular siblings, at ~0.85 compared to ~0.50, constitutes further evidence for genetic effects. An environmental model would require that this difference is fully due to differences in environmental pressures toward similarity. We also found that the correlations for 1st cousins and 2nd cousins were higher if they were related through same-sex twins of unknown zygosity, to an extent that is consistent with substantial genetic effects.

Twins are then an important potential exception to our suggestion that genetic and environmental effects have similar patterns of correlations across categories of biological relatives: monozygotes are genetically identical while dizygotes are not, but most twin pairs, regardless of zygosity, grow up together in the same house. The predicted gap between rMZ and rDZ would then narrow if genetic effects are substituted for shared-environmental effects. Above, we argued that shared-environmental effects are necessary to appropriately model relatives-in-law and adoptive relatives. The rMZ–rDZ gap is too narrow even in our model with no shared-environmental effects at all, however, so every such step toward making the model fit better with relatives-in-law and adoptive relatives would exacerbate this problem.

There are several potential ways to explain elevated values of rMZ relative to rDZ, which are not mutually exclusive. For example, certain kinds of gene–environment interplay can have this effect (25, 26). Nonadditive genetic effects, such as dominance effects or epistasis, can also contribute: these effects are less strongly correlated between DZ twins than what additive genetic effects are, yet still perfectly correlated between MZ twins. Environmental effects could also serve to explain large rMZ–rDZ gaps, if they have this same kind of pattern of being much more strongly correlated between monozygotes than dizygotes (i.e. if the “equal environments assumption” is severely wrong). All these effects do little or nothing to increase similarity between nonimmediate relatives, and they would thus have minimal influences on parameter estimates in Fisherian models on samples without MZ twins.

Interestingly, in a classical twin design, our twin correlations imply that 75% of phenotypic variance is due to additive genetic effects, and that 11% is due to shared-environmental effects. This model predicts rMZ and rDZ perfectly, and it also gives quite accurate predictions for adoptive siblings, via the shared-environmental effect. From the perspective of the twin model, it is rather the high correlations between more distant relatives that need an explanation. The classical twin design assumes random mating, so expected genotypic correlations between distant relatives would be low: Their high phenotypic correlations would then need to be explained almost entirely with shared-environmental effects. It is increasingly clear, however, that mating is not random for educational outcomes (27). If we make a more plausible assumption about the genotypic correlation of mates than zero, such as 0.25, then the shared-environmental effects would disappear. The estimated *h*^2^ would increase to 0.86, and we would essentially have a model giving similar, but less accurate, predictions for nonimmediate relatives to those of our Fisherian models.

### Limitations and Future Directions.

A limitation of this study is its generalizability. While our sample is highly representative of the population of school students in Norway between 2007 and 2019, as it contains almost all of them, it is not necessarily representative of other countries or time periods. Nor do our results necessarily generalize to adults. In the case of adoptive siblings, for example, it is important to be aware of evidence that the strength of their correlation on cognitive ability decreases as they grow older (28, 29).

Another limitation concerns dependencies between observations, as many individuals have several relatives in our data. As is also the case in, e.g., Clark (8), we do not account for these dependencies, so the CI around our correlations are likely to be too narrow. When comparing our estimates to those from the approach used in Hällsten (30), which fully accounts for dependencies and which can be applied to a subset of our correlations, we find that CI widen by less than 30% (*SI Appendix*, section 3.2 and Tables S4 and S5).

The continued exploration of the mechanisms behind assortative mating (14, 31) is consequential to how our correlations are to be interpreted. Our modeling approach assumes that mates are assorting directly or indirectly on traits that influence test scores, and that the level of assortment has been constant across generations and has now reached equilibrium (32). If these assumptions prove inappropriate, then models that do not require them are preferable.

Another notable assumption inherent in our approach is that the mere fact of being identified as having a relative of a certain kind is uninformative about one’s phenotype (33). *SI Appendix*, Table S10 shows averaged test scores for subsets of students identified as having particular categories of relatives: Most of these averages are close to the full sample average, with the largest differences being for students with half siblings and students with step siblings, with averages that are 0.17 and 0.28 SD lower than that of the full sample, respectively.

We hope to see the development of models with both genetic and environmental sources of family resemblance that can be fitted to datasets such as ours. This would require a framework with environmental effects that does what the Fisherian framework does for genetic effects, which is to describe how they are predicted to correlate across relative types under assortative mating.

As pointed out by Young (34), the introduction of molecular genetic data into analyses of correlations between distant relatives could improve our ability to distinguish genetic and cultural transmission, taking advantage of how genetic material is randomized in meiosis.

It could also be fruitful to use large registry data to investigate the sources of environmental effects, while controlling for genetic effects. The present study only contains indications that there are environmental effects on family resemblance, while saying very little about the nature of these effects. This would require detailed information about households, neighborhoods, schools, and so on.

## Conclusions

We document substantial family resemblance on educational performance, as measured by Norwegian national standardized tests, across a wide range of categories of relatives. In explaining our set of correlations, we find that Fisherian models, which posit additive genetic effects and assortative mating as the only sources of similarity between relatives, are insufficient. The models’ inability to explain high correlations between adoptive relatives, between relatives-in-law, and between maternal relatives, suggests the presence of environmental sources of family resemblance.

We also see a need for ways to account for the strong effects of zygosity on correlations between twins, beyond just additive genetic effects. Potential candidates here include nonadditive genetic effects, and gene–environment interplay. Our study then serves as further cause for caution when interpreting estimates from twin models. If such effects selectively increasing rMZ coexist with shared-environmental effects, which increase all twin correlations equally, then rMZ could end up about twice as high as rDZ so that a twin model concludes that neither of the effects exist and that all twin similarity is due to additive genetic effects.

While we here show that Fisherian models appear not to be sufficient, we have not offered any precise specifications of models that are. By publicly sharing our correlation data through this paper, we enable other researchers to fit and evaluate alternative models of their choosing. We are hopeful this will contribute toward further improvements to our understanding of the causes of family resemblance in the future.

## Materials and Methods

Since 2007, Norwegian school children have been required to take standardized tests, in math and in Norwegian and English reading comprehension. These tests are given in the 5th, 8th, and 9th grade (with no English test in the 9th grade), which are the years students turn 10, 13, and 14 y old, respectively. In Norway, the time for starting school is determined by one’s birthyear, so, with very few exceptions, students in the same grade are born in the same year.

The tests are taken by over 95% of children going through the Norwegian school system (15). Only students receiving special education and students who do not have Norwegian as their first language can get an exemption, if they apply for it.

The tests in math and Norwegian reading have time limits of 90 min, while the English test has a limit of 60 min. They all consist of ~40 questions each. New tests are developed for each year, with comprehensive work using item response theory going into ensuring that results are comparable from year to year, and that the tests are sensitive to individual differences across the entire range of ability (15).

The stated purpose of the tests is “to measure fundamental abilities in reading, math and English that are important for learning across all subjects” (15). They do not measure knowledge of any curriculum, so it is not possible, nor encouraged, for students to study for the tests. Scores on the different tests, whether looking across grades or subjects, have high within-person correlations, ranging from 0.46 to 0.86 (*SI Appendix*, Table S1).

The present study was approved by The Regional Committees for Medical and Health Research Ethics (Project No. 2017/2205) “Social Inequality in Norway and Children’s Psychological Development.” The committee waived the requirement for informed consent due to the use of deidentified administrative data.

### Sample.

Our sample consists of everyone who took Norwegian national tests between their implementation in 2007 and up until 2019, N = 936,708 (93% of whom, 866,381, have at least one identified relative in the sample). Of these, 700,173 have scores for the 5th grade tests, 719,984 have scores for the 8th grade tests, and 537,646 have scores for the 9th grade tests. 448,381 students have scores for the tests in all three grades (*SI Appendix*, section 4 and Table S7 have further information about test attendance). Table 1 shows numbers of pairs for each category of relative where both individuals in the pair have completed tests in the 8th grade. Corresponding numbers for the 5th and 9th grade tests are in *SI Appendix*, Table S6.

#### Identification of relatives.

Since 1963, Norway has registered all its residents in a population registry for administrative purposes. We have access to an anonymized version of the registry, where hashed identification codes for each person are connected to the codes of their parents, thereby allowing us to identify pairs of relatives. Twins are identified by matching siblings on birthdates. For a subset of our 9,390 pairs of same-sex twins, we have access to information about their zygosity, either through the Norwegian twin registry (35) (NTR; 1,302 pairs) or the Norwegian Mother and Child Cohort Study (36) (MoBa; 783 pairs). For same-sex twins who are not part of twin registries, we leave their zygosity as unknown (7,305 pairs). Opposite sex twins are always dizygotic (5,371 pairs).

In cases where two people are connected through a single shared ancestor and this person’s mate is missing for both of them, we assume that this missing person is the same person in both cases (*SI Appendix*, section 3.1 and Table S3). For example, pairs connected through a grandmother whose mate is missing for both of them will be assumed to be 1st cousins instead of half 1st cousins.

Individuals are inferred to be adopted if they are born in one of the countries Norwegians were able to adopt from in the relevant time period, and both their parents are born in Norway. Out of the 7351 such children in our sample, 31% were born in South Korea, 19% in Columbia, 19% in China, 7% in Ethiopia, and the remaining 23% divided between 14 other countries.

In total, our sample then has the following numbers of pairs: 422,339 full siblings (including 14,761 twin pairs), 89,643 half siblings, 1,355,741 1st cousins (including 10,904 pairs related through same-sex twins), 125,404 half 1st cousins, and 2,146,011 2nd cousins (including 15,679 pairs related through same-sex twins), 17,488 step siblings, and 1,164,933 co-cousins. Additionally, we have pairs of relatives through adoption: 3,340 siblings, 23,407 1st cousins, 23,531 2nd cousins, and 23,447 co-cousins.

### Analysis.

Prior to calculating correlations, we adjust test scores to account for the fact that tests are continuously updated from year to year. To make scores more comparable across years, we convert scores to percentile ranks relative to the other students taking the same test that year, and then convert these to z-scores. We also control for age differences between students taking the same test, by regressing out effects of birth month. For adoptees, we control for effects of their country of origin, by using residualized scores from a regression predicting test scores from country of origin, within the sample of adoptees. We also make available correlations between adoptive relatives that are uncorrected for country of origin.

#### Modeling approach.

If family resemblance is fully explained by additive genetic effects and assortative mating, then phenotypic similarity is purely a function of genotypic similarity. Under random mating, first degree relatives have an expected genotypic similarity of 0.50, reflecting the fact that, on average, 50% of their genes are identical by descent (IBD). As relatives become more distant, the expected proportion of genes being IBD is continuously halved, since parents pass on exactly half their genome to each child. If genes affect traits only additively, then phenotypic similarity for *n*th degree relatives, $p_{n}$, will be a proportion of genotypic similarity that is equal to the trait’s heritability, $p_{n}\;=\;h^{2}{\left(\frac{1}{2}\right)}^{n}$.

Assortative mating can cause the genotypic similarity of relatives to increase. If mates are assorting on a heritable trait, then they will be more likely to be genotypically similar on that trait, not in the sense that any of their genes are IBD, but rather that the effects of their genes are more likely to go in the same direction. Their children will then be more genotypically similar to each other than otherwise, because also the genes that are not IBD between them will be correlated in how they affect the relevant phenotype. This increased similarity between siblings will then have ripple effects throughout the family tree, particularly if these siblings and their descendants also mate assortatively. Over the last century, the theoretical consequences of assortative mating on family resemblance have been laid out (10, 21, 32, 37). In the Fisherian framework, the formula for predicted phenotypic correlations for full siblings and *n*th degree relatives related through full siblings, is updated by the addition of a term *m*, which represents the genotypic correlation between mates for the relevant trait:

$$p_{n}=h^{2}{\left(\frac{1+m}{2}\right)}^{n}.$$

Expected correlations for other common categories of relatives build from this formula. In the case of half siblings, and pairs related through half siblings, Nagylaki (38) showed that the expression needs to be multiplied by $\frac{1+2m+rm}{{\left(1+m\right)}^{2}}$, where *r* is the phenotypic mate correlation. This will appropriately adjust for the fact that, under assortative mating, half siblings will be genotypically similar not only through the parent they share but also through the parents they do not share, because the unshared parents will both tend to be similar to the shared parent. As Yengo and Visscher (39) point out, this multiplier tends to be close to 1, more so the closer *r* is to *m*.

To get expectations for relatives-in-law, chains of biological relatives can be linked together by multiplying with *m*. Then, *n* is no longer the degree of relatedness, but rather the combined relatedness the relatives-in-law have to their connecting pair (step siblings have *n* = 2, for example, because this is the sum of the two *n* = 1 paths they have to their parents). *SI Appendix*, Table S9 has an overview of expressions for predicted correlations.

Categories of relatives for which the degree of relatedness, *n*, depends on an unknown zygosity in a twin pair, are not part of our model fitting. Predictions for these categories can still be calculated once *h*^2^ and *m* have been estimated, however, if an assumption is made about the proportion of same-sex twins who are monozygotic. For the predictions in our tables and figures, we set this proportion to be 0.50. Ratios of same-sex to opposite sex pairs among twins in our registry data suggest that this is accurate for 1st and 2nd cousins connected through twins, but that the proportion of monozygotes has decreased in recent years (to ~42%), potentially due to increased use of in vitro fertilization.

In order to fit a model based on this framework, we follow the approach used by Clark (8), and use the natural logarithms of our correlations as our dependent variable, rather than the correlations themselves. The expressions then all take a form that is more amenable to analysis, through simple linear regression. For full siblings and their descendants, $p_{n}\;=\;h^{2}{\left(\frac{1+m}{2}\right)}^{n}$ implies that:

$$\ln(p_{n})=\ln(h^{2})+n*\ln\left(\frac{1+m}{2}\right).$$

For half siblings and their descendants, $p_{n}\;=\;h^{2}{\left(\frac{1+m}{2}\right)}^{n}\frac{1+2m+rm}{{\left(1+m\right)}^{2}}$ implies that:

$$\ln(p_{n})=\ln(h^{2})+n*\ln\left(\frac{1+m}{2}\right)+\ln\left(\frac{1+2m+rm}{(1+m{)}^{2}}\right).$$

For relatives-in-law, $p_{n}\;=\;h^{2}m{\left(\frac{1+m}{2}\right)}^{n}$ implies that:

$$\ln(p_{n})=\ln(h^{2})+n*\ln\left(\frac{1+m}{2}\right)+\text{ln}\left(m\right).$$

We can then estimate *h*^2^, *m,* and the value of the multiplier in the expression for relatives through half siblings, from the parameters of a linear regression:

$$\text{ln}\left(p_{n}\right)=b_{0}+b_{1}n+b_{2}d_{\mathrm{hs}}+b_{3}d_{\mathrm{law}}+\varepsilon .$$

$b_{0}$ then estimates ${ln(h}^{2})$, and $b_{1}$ estimates $\text{ln}\left(\frac{1+m}{2}\right)$. We can then calculate *h*^2^ and *m* from the estimates of $b_{0}$ and $b_{1}$. $d_{\mathrm{hs}}$ is an indicator variable for pairs who are half siblings or half 1st cousins: $b_{2}$ is then an estimate of the logarithm of the multiplier in the formula for these pairs, which is $\frac{1+2m+rm}{{\left(1+m\right)}^{2}}$. We refer to the estimated value of this multiplier as *c*. $d_{\mathrm{law}}$ is an indicator variable for pairs who are relatives-in-law: $b_{3}$ is then an estimate of $\ln\left(m\right).$ A constraint is added such that the values of *m* implied by $b_{1}$ and $b_{3}$ are the same. The model is fitted using weighted least squares estimation, where each correlation is weighted by the inverse of its squared SE, so that correlations estimated with more precision have a higher impact on parameter estimates and R^2^.

#### Analysis of effects of gender composition.

To investigate effects of the gender composition of pairs, we ran a linear regression predicting our correlations from an indicator variable valued 1 for same-sex pairs and 0 otherwise, while controlling for the category of relative to reduce unexplained variance, and weighting correlations by their precision. We also ran the same kind of analysis on only correlations for same-sex pairs, predicting correlations from whether they are for male or female pairs. The indicator variable was then instead for whether the pair was male or female.

## Supplementary Material

Appendix 01 (PDF)

## Data Availability

Anonymized (sets of correlations) data have been deposited in Open Science Framework (https://doi.org/10.17605/OSF.IO/JFW2K) (19).
