# Supplementary material for: Beyond additive genetic effects: Explaining family resemblance in school performance across millions of pairs of Norwegian relatives
Source: Proc Natl Acad Sci U S A. 2025 Jun 20;122(25):e2419627122. doi: 10.1073/pnas.2419627122 (PMC12207517; doi:10.1073/pnas.2419627122)
Supplement: Supplementary file 1 — Appendix 01 (PDF) [file pnas.2419627122.sapp.pdf]

Supporting Information for

Beyond additive genetic effects: Explaining family resemblance in school performance across millions of pairs of Norwegian relatives

Nikolai Haahjem Eftedal[1]*
Espen Moen Eilertsen[1]
Hans Fredrik Sunde[2]
Thomas Haarklau Kleppestø[3]
Eivind Ystrom[1,4]
Nikolai Olavi Czajkowski[1,4]

1Promenta Research Centre, Department of Psychology, University of Oslo, Oslo, Norway
2Centre for Fertility and Health, Norwegian Institute of Public Health, Oslo, Norway
3Department of Psychology, Norwegian University of Science and Technology, Trondheim, Norway
4PsychGen Centre for Genetic Epidemiology and Mental Health, Norwegian Institute of Public Health, Oslo, Norway.

*To whom correspondence may be addressed: **Email:** n.h.eftedal@psykologi.uio.no

This PDF file includes:

Supporting text
Figure S1
Tables S1 to S10
SI References

Supporting Information Text

1. Description of datasets

In our main text, we present 82 separate correlations between relatives on the averaged score on the three tests taken in the 8th grade. At https://osf.io/jfw2k/, there are files containing the corresponding correlations for several more phenotypes. There are eight different national tests each student takes as part of going through the Norwegian school system: there are three subjects – Norwegian and English reading comprehension, and Mathematics – and there are separate tests for the 5th, 8th, and 9th grade. As there is no English test in the 9th grade, this adds up to eight. We offer files containing correlations for the 82 categories of relatives for each single test. Additionally, we have files with averaged scores on the three tests in the 5th grade, the three tests in the 8th grade, and the two tests in the 9th grade (with separate files for correlations with and without exclusion of students who did not take all three tests). We also have files with just the average of the math and norwegian tests in the 5th and 8th grade, to make correlations more directly comparable across the 5th, 8th, and 9th grades (since there is no english test in the 9th grade). To allow researchers some freedom to choose how to deal with effects of country of origin of adoptees, we offer files with correlations between adoptive relatives in their uncorrected form, which can then be combined with the other files if desired.

An R-script for our analyses is also available at https://osf.io/jfw2k/, named “analysis”.

Files with single subject correlations are named “math”, “read”, or “eng”, depending on whether they are for math, Norwegian reading comprehension or English reading comprehension, respectively. Whether tests are for 5th, 8th, or 9th grade is indicated by a number following the name. “eng8” is then correlations for the english tests in the 8th grade, for example. Files containing correlations for averaged scores for several subjects, are named either “MRE” if they are for all three subjects, or “MR” if they are for math and reading only. Files with the correlations for adoptive relatives that are uncorrected for country of origin follow the same naming scheme but with “_ado_uncorr” added at the end.

The files have the following columns:

- **corr**: The estimated correlation
- **SE**: The standard error of the estimate of the correlation
- **rel**: The category of relative the correlation is for. The categories are:
    - **mz**: monozygotic twins
    - **dz**: dizygotic twins
    - **uz**: for same-sex twins of unknown zygosity
    - **sibs**: siblings who are not twins
    - **half sibs**: half siblings
    - **1st cousins t/uz**: 1st cousins related through same-sex twins of unknown zygosity
    - **1st cousins**: 1st cousins not related through same-sex twins
    - **half cousins**: Half 1st cousins
    - **2nd cousins t/uz**: 2nd cousins related through same-sex twins of unknown zygosity
    - **2nd cousins**: 2nd cousins not related through same-sex twins
    - **step sibs**: Pairs not related by blood who have one or more half siblings in common
    - **cocousins**: Pairs not related by blood who have one or more cousins in common
    - **adoptive sibs**: Siblings where at least one is adopted from outside Norway
    - **adoptive 1st cousins**: 1st cousins where at least one is adopted from outside Norway
    - **adoptive 2nd cousins**: 2nd cousins where at least one is adopted from outside Norway
    - **adoptive cocousins**: cocousins where at least one is adopted from outside Norway
- **type**: Takes the value **maternal** for relatives related through their mothers, **paternal** for relatives related through their fathers, and **other** for pairs of relatives who cannot be categorized as maternal or paternal
- **pair**: Valued **ssf** for same-sex female, **ssm** for same-sex male, and **os** for opposite sex pairs
- **half**: Indicator for half relatives, valued 1 for half siblings and half 1st cousins, and 0 otherwise

- **npairs**: Number of pairs of relatives in the category for that row
- **weight**: Inverse of the squared standard error
- **ns**: For biological relatives, this is their degree of relatedness (for UZ twins and relatives through UZ twins, where relatedness depends on an unknown zygosity, it takes the average of the two alternatives). For relatives-in-law, the value represents the combined distance to their connecting pair of mates.
- **ril**: Indicator for relatives-in-law, valued 1 for step siblings, co-cousins, and adoptive co-cousins, and 0 otherwise
- **ado:** Indicator for adoptive relatives, valued 1 for adoptive siblings, adoptive 1st cousins, adoptive 2nd cousins, and adoptive co-cousins, and 0 otherwise.
- **log_corr:** The natural logarithm of the values in the corr column

2. Analyses on separate test scores, and on several averages of test scores

2.1 Fisherian models

Table S2 shows estimates of h^2, m, and c, for each of the separate national tests, as well as for averaged scores on the tests in the 5th grade and in the 9th grade. h^2 is heritability, m is genotypic partner correlation, and c is the estimated value of the ratio in the expressions for predicted phenotypic correlations for half siblings and descendants of half siblings. R^2's are also included. All estimates are made using the same modelling approach we used in the main text for the averaged score on the 8th grade tests. Additionally, we also show these same estimates and R^2's for when models are fitted to only correlations between biological relatives.

Estimates of h^2 range from .347 to .506, and estimates of m range from .340 to .439. For the models on only biological relatives, estimates of h^2 are substantially higher, ranging from .613 to .801, and estimates of m are substantially lower, ranging from .116 to .246. R^2's are also highest among the models on only biological relatives, where they range from .926 to .992, as compared to the models also fitted to correlations between in-laws, where R^2s range from .809 to .885.

2.2 Twin models

Table S2 also contains variance components from the classical twin design, where we estimate *A*, *C*, and *E* from just the correlations between monozygotic and dizygotic twins. *A* is additive genetic effects, *C* is shared-environmental effects, and *E* is non-shared-environmental effects. Here, we see quite high heritabilities, ranging from .540 to .787, and we see small shared-environmental effects ranging from .035 to .200.

3. Investigating assumptions and limitations

3.1 Assumption regarding missing data when inferring relatedness

The registry contains ID codes of a person's mother and father. These connections between parents and children are our basis for identifying pairs of relatives. Sometimes these codes are missing. This requires us to make certain assumptions when inferring relatedness. For example, let us say that two people have the same mother in the registry and that they both have missing data for their father. Then, this pair could be full siblings, if they have the same father, or they could be half siblings, if they have different fathers. A similar situation arises when two people share a parent, but one of them misses the ID of the other parent while the other does not. Again, they could be either half siblings or full siblings, depending on the identity of the missing person.

Our choice for how to handle these issues is to assume that if someone has a missing partner for several of their children, this is always the same partner. And if someone has a missing partner for some of their children, but not for others, then the missing person is different from the person who is not missing (when people are missing from the registry this is often because they died before registration started, in 1964, or they never lived in Norway; for both of these cases, they would then be missing for all of their children, not just some of them). With these assumptions, people who share a parent and who both have missing IDs for the other

parent are assumed to be full siblings, while people who share a parent and where one has a parent missing and the other does not will be assumed to be half siblings. Inferences about the relatedness of the descendants of such pairs will then be affected accordingly.

The most common alternative to our approach here is to assume that people only share the ancestors that are registered, and that whenever an ancestor is unregistered, this ancestor is not shared with anyone else. Table S3 compares our correlations to the ones we would get with this alternative approach. As can be seen, differences are generally minimal. The most important points are that several pairs we see as full siblings are here considered half siblings (most commonly maternal, since it is rare to be missing one's mother but not one's father), that some 1st cousins get categorized as half cousins, and that several 2nd cousins are no longer identified as such (i.e. our numbers of pairs of 2nd cousins are decreased). The correlations between "full" relatives remain largely unchanged, suggesting that most of the pairs we assumed to be full relatives were indeed full relatives. Correlations between half relatives, particularly half siblings, increase a little bit, suggesting that many of the new pairs categorized as half relatives are in reality full relatives (i.e. they were indeed what we originally categorized them to be).

3.2 Pseudo replications

As detailed in Table S4, there are many people in our data who are related to several others who are also in our data. This creates an issue known as pseudo replication[1], where many individuals are part of several of our observations, thus making dependencies between them. This issue is more pronounced for correlations between more distant relatives. It does not exist at all for twins, where all our twins are part of just one twin pair. But for 2nd cousin pairs, our average subject has more than three 2nd cousins who are also in the sample, and a few have more than a hundred.

We do not account for these dependencies, which can cause our confidence intervals to be too narrow. We did not find a method of accounting for this issue which could be applied to all the correlations we want to estimate without creating its own set of problems. However, the method applied in Hällsten[2], which is again based on Solon, Page, and Duncan[3], can be applied to a subset of our correlations. This approach (which is described in more detail in the originating works) involves calculating intraclass correlations between people sharing ancestors, in a multilevel model, with weighting to account for how extended families can vary in size. Table S5 compares standard errors for correlations from this approach to ones without correction for pseudo replications, for siblings, maternal and paternal 1st cousins, and matrilineal and patrilineal 2nd cousins. We find that standard errors are indeed larger, but none of them by more than 30%.

4. Test attendance

For the correlations in the main text, we use as our phenotype the average of the tests a student has taken in the 8th grade. For 92% of the students who have taken any of the three tests in the 8th grade, this will be all three tests. But some students missed either one or two of the tests (6% and 2%, respectively), so their scores are then the average of only the tests they have taken. If we had instead only included students who had completed all three tests, this would have not appreciably changed any of our results or conclusions. We include among the sets of correlations we make available on OSF correlations of averages of the tests in each grade both with and without exclusion of students who did not complete all the tests in the relevant grade.

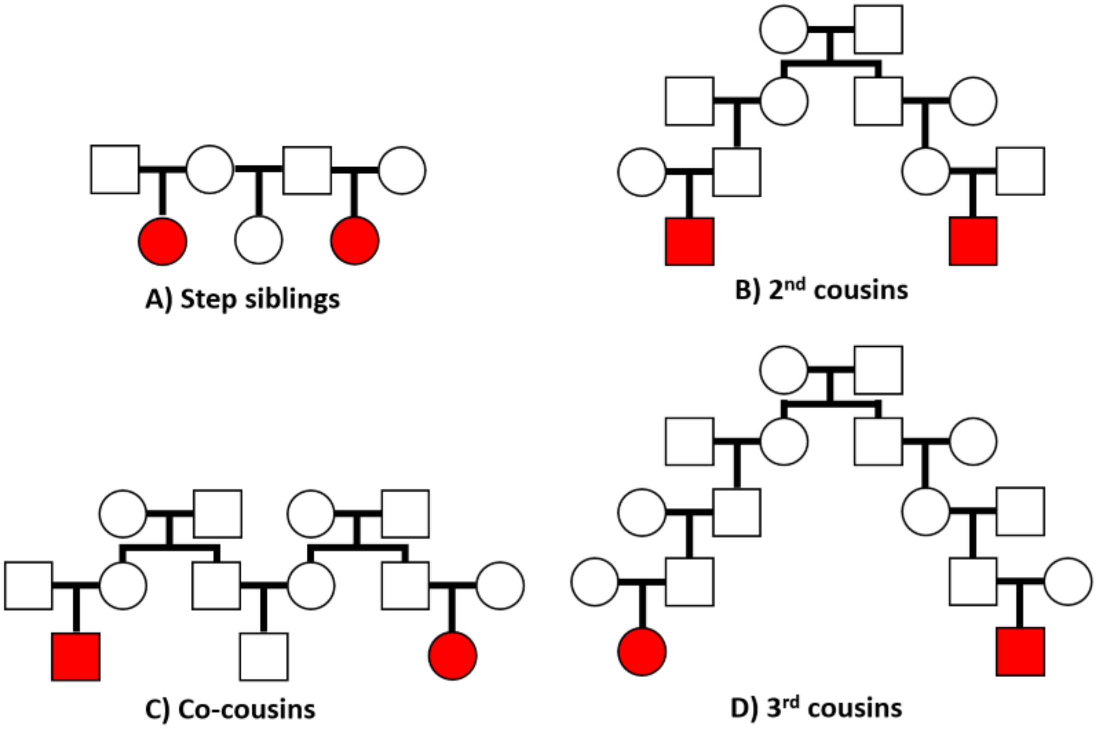

Fig. S1. Pedigree drawings of step siblings (S1A), 2nd cousins (S1B), co-cousins (S1C), and 3rd cousins (S1D). The drawings are meant to illustrate that, in terms of distance in a pedigree, step siblings are closer than 2nd cousins (three steps needed to get from one to the other for step siblings versus five steps needed for 2nd cousins), and co-cousins are closer than 3rd cousins (five steps needed for co-cousins versus seven steps needed for 3rd cousins). As table S8 shows, step siblings and 2nd cousins are about equally distant genotypically, for plausible values of genotypic partner similarities, and so are co-cousins and 3rd cousins.

Table S1. Correlations between scores on the separate tests taken by the same person

	Eng 5th	Rea 5th	Mat 5th	Eng 8th	Rea 8th	Mat 8th	Rea 9th
Rea 5th	.627						
Mat 5th	.527	.622					
Eng 8th	.718	.648	.504				
Rea 8th	.575	.709	.588	.712			
Mat 8th	.483	.580	.761	.582	.680		
Rea 9th	.548	.680	.571	.683	.797	.661	
Mat 9th	.461	.561	.739	.560	.652	.856	.681

Note. Rea is norwegian reading comprehension, Eng is english reading comprehension, Mat is mathematics, 5th is for 5th grade tests, 8th is for 8th grade tests, 9th is for 9th grade tests.

Table S2. Estimated parameters, from Fisherian and twin models, on all our phenotypes

	Fisherian, bio + in-law				***Fisherian, bio only***				***Twin models***		
	Estimates				*Estimates*						
**Test**	h^2	*m*	*c*	R^2	h^2	*m*	*c*	R^2	*A*	*C*	*E*
Math, 5th	.397	.396	0.91	.862	.623	.215	0.92	.982	.664	.119	.217
Reading, 5th	.383	.390	0.93	.879	.613	.196	0.94	.983	.548	.151	.301
English, 5th	.347	.340	0.98	.809	.642	.116	0.96	.982	.540	.200	.260
Math, 8th	.464	.424	0.92	.877	.710	.236	0.95	.978	.787	.035	.178
Reading, 8th	.427	.402	0.98	.850	.762	.161	1.00	.943	.664	.078	.257
English, 8th	.430	.385	1.00	.850	.734	.164	1.02	.991	.691	.115	.194
Math, 9th	.458	.422	0.91	.870	.695	.240	0.94	.979	.778	.032	.189
Reading, 9th	.428	.389	0.91	.848	.751	.157	0.96	.926	.649	.073	.279
MRE, 5th	.427	.407	0.95	.859	.688	.216	0.95	.991	.612	.194	.194
MRE, 8th	.503	.430	0.97	.873	.801	.225	1.00	.988	.752	.111	.137
MR, 5th	.447	.411	0.91	.885	.681	.236	0.92	.992	.618	.179	.202
MR, 8th	.506	.438	0.94	.885	.777	.245	0.97	.987	.729	.109	.162
MR, 9th	.494	.439	0.91	.873	.766	.246	0.95	.986	.717	.104	.179

Note. *MRE is the average of tests in Math, Norwegian Reading, and English. MR is the average of tests in Math and Norwegian Reading.* h^2 *is heritability, m is genotypic partner correlation, c is the value of the ratio in the expressions for predicted phenotypic correlations for half siblings and descendants of half siblings. Columns under "Fisherian, bio + in-law" show information about models fitted to correlations for all our biological relatives and also step siblings and co-cousins. Columns under "Fisherian, bio only" are for models fitted to correlations for biological relatives only. For the the twin models, A, C, and E are the additive genetic, shared-environmental, and unique-environmental variance components, respectively.*

Table S3. Correlations on 8th grade tests with different assumptions about missing data

Relation	N, Asmpt. 1	N, Asmpt. 2	r, Asmpt. 1	r, Asmpt. 2
MZ twin	795	795	.863	.863
UZ twin	5,648	5,648	.662	.662
DZ twin	5,116	5,116	.487	.487
Sibling	285,025	282,191	.481	.479
Half sibling – mat	25,891	28,713	.290	.317
Half sibling – pat	22,942	23,268	.239	.245
1st cousin t/ UZ twins	7,606	7,547	.258	.259
1st cousin – mat	240,187	239,527	.203	.203
1st cousin – pat	237,489	236,763	.188	.188
1st cousin – opp	455,840	454,733	.182	.181
Half 1st cousin – mat	21,132	21,962	.115	.117
Half 1st cousin – pat	17,048	18,347	.131	.140
Half 1st cousin – opp	37,695	39,264	.126	.127
2nd cousin t/ UZ twins	9,100	9,020	.107	.106
2nd cousin – mat	352,298	321,289	.075	.073
2nd cousin – pat	249,651	218,567	.066	.063
2nd cousin – opp	577,694	517,637	.068	.066
Step sibling	10,167	10,167	.127	.127
Co-cousin – mat	221,595	221,498	.076	.076
Co-cousin – pat	186,182	186,128	.074	.074
Co-cousin – opp	401,159	401,117	.074	.074
Adopted sibling	2,536	2,536	.149	.149
Adopted 1st cousin	17,361	17,343	.045	.045
Adopted 2nd cousin	15,230	11,826	.014	.017
Adopted co-cousin	17,358	17,355	.042	.042

Note. All correlations are for the averaged score on the three tests in the 8th grade. Asmpt. 1 is the assumption we use, where siblings are assumed to share both parents if they have matching IDs for one parent and both have missing IDs for their other parent. Asmpt. 2 is when such siblings are instead inferred to be half siblings (i.e. that their missing parents are assumed to be different people). N is the number of identified pairs, r is observed correlations.

Table S4. Numbers of other relatives in the sample, for the 8th grade tests

Relation	Mean	Max	> 0	Mean, >0
Siblings	0.83	8	62%	1.35
Half siblings	0.14	9	10%	1.38
1st cousins	2.64	100	73%	3.61
Half 1st cousins	0.21	21	9%	2.29
2nd cousins	3.48	142	51%	6.35

Note. *"Mean" is the mean number of identified relatives in a category an individual has within the sample (consisting of individuals with valid scores on the 8th grade national tests). "Max" is the highest observed number of relatives connected to a single person for each category. ">0" is the percentage of the sample with at least one relative in the relevant category. "Mean, >0" is the mean number of relatives in the relevant category among only the subset of the sample with at least one relative in that category.*

Table S5. Standard errors with and without correction for pseudo replications

Relation	SE, no correction	SE, Solon
Sibling	.0016	.0017
1st cousin – mat	.0020	.0023
1st cousin – pat	.0020	.0025
2nd cousin – mat	.0033	.0042
2nd cousin – pat	.0038	.0046

Note. *"SE, no correction" are standard errors calculated with no correction for pseudo replications. "SE, Solon" are correlations calculated using the approach in Hällsten[2], which is again based on Solon, Page, & Duncan[3], which corrects for pseudo replications. "mat" and "pat" here mean "matrilineal" and "patrilineal", such that the entire connecting chain between a pair of relatives must all have the same gender. For 1st cousins, matrilineals are connected through sisters and patrilineals are connected through brothers (so the terms imply the same as "maternal" and "paternal" here). For 2nd cousins, matrilineals are connected through a same-sex female pair of maternal 1st cousins (such that only about 1 in 16 pairs of 2nd cousins are matrilineal). Patrilineal 2nd cousins are connected through a same-sex male pair of paternal 1st cousins.*

Table S6. Numbers of pairs of relatives

Relation	N pairs, total	N pairs, 5th	N pairs, 8th	N pairs, 9th
MZ twin	942	704	795	573
UZ twin	7,305	5,747	5,648	4,302
DZ twin	6,514	5,125	5,116	3,894
Sibling	407,634	270,106	285,025	173,862
Half sibling – mat	46,674	23,867	25,891	10,764
Half sibling – pat	42,841	21,112	22,942	9,502
1st Cousin t/ UZ twins	10,904	7,303	7,606	4,667
1st Cousin – mat	347,389	212,987	240,187	135,594
1st Cousin – pat	345,276	211,347	237,489	133,793
1st Cousin – opp	660,724	403,673	455,840	257,172
Half 1st cousin – mat	35,017	21,631	21,132	11,736
Half 1st cousin – pat	27,995	17,741	17,048	9,517
Half 1st cousin – opp	62,014	38,704	37,695	20,907
2nd cousin t/ UZ twins	15,679	11,493	9,100	5,417
2nd cousin – mat	609,813	427,191	352,298	206,814
2nd cousin – pat	441,538	313,784	249,651	146,147
2nd cousin – opp	1,014,454	714,846	577,694	388,404
Step sibling	17,488	12,289	10,167	5,832
Co-cousin – mat	318,187	186,220	221,595	121,656
Co-cousin – pat	269,145	158,136	186,182	102,563
Co-cousin – opp	577,601	338,647	401,159	220,747
Adopted sibling	3,284	2,187	2,536	1,655
Adopted 1st cousin	23,407	14,256	17,361	10,302
Adopted 2nd cousin	23,531	16,165	15,230	9,163
Adopted co-cousin	23,447	13,881	17,358	10,394

Note. *N pairs total is the total numbers of identified pairs of relatives for each category in our sample. N pairs, 5th, 8th, and 9th are the numbers of pairs where both have valid scores for the set of tests in the specified grade.*

Table S7. Test attendance; numbers taking zero, one, two, or three tests, for each grade

	Tests taken			
**Grade**	**0**	**1**	**2**	**3**
5th	236,535	14,430	82,633	603,110
8th	216,724	10,995	45,488	663,501
9th	399,062	28,111	509,535	-

Note. For each grade, we here show how many students in our sample who have taken 0, 1, 2, or 3 of the tests given in that grade. The numbers in the 0 tests taken category include students who had simply not reached that grade yet at the time our sample was gathered, or who reached that grade before national tests started in 2007. There is no test in English in the 9th grade, so the maximum number of tests to take in the 9th grade is 2

Table S8. Expected genotypic correlations, for step siblings, 2nd cousins, co-cousins, and 3rd cousins, under different levels of assortment

Relation	Predicted r_g	*m* .00	.10	.20	.30	.40	.50	.60
Step sibling	$m\left(\frac{1+m}{2}\right)^2$	.000	.030	.072	.127	.196	.281	.384
2nd cousin	$\left(\frac{1+m}{2}\right)^5$	.031	.050	.078	.116	.168	.237	.328
Co-cousin	$m\left(\frac{1+m}{2}\right)^4$	.000	.009	.026	.054	.096	.158	.246
3rd cousin	$\left(\frac{1+m}{2}\right)^7$	.008	.015	.028	.049	.082	.133	.210

Note. Relative type is the category of relative; Predicted r_g is the expressions for predicted genotypic correlations in the Fisherian framework; *m* is the genotypic partner correlation. As can be seen, values for step siblings are quite similar to those of 2nd cousins, and values of co-cousins are quite similar to those of 3rd cousins, particularly when *m* is between .20 and .40.

Table S9. Parametric specifications of expected familial correlations

Relation	Predicted correlation
Monozygotic twin	h^2
Same-sex twin, unknown zygosity	$h^2\left[p_{mz}\left(\frac{1+m}{2}\right)^0 + p_{dz}\left(\frac{1+m}{2}\right)^1\right]$
Dizygotic twin | Full sibling	$h^2\left(\frac{1+m}{2}\right)^1$
Half sibling	$h^2\left(\frac{1+m}{2}\right)^2 \frac{1+2m+rm}{(1+m)^2}$
1st cousin through same sex twins with unknown zygosity	$h^2\left[p_{mz}\left(\frac{1+m}{2}\right)^2 + p_{dz}\left(\frac{1+m}{2}\right)^3\right]$
1st cousin	$h^2\left(\frac{1+m}{2}\right)^3$
Half 1st cousin	$h^2\left(\frac{1+m}{2}\right)^4 \frac{1+2m+rm}{(1+m)^2}$
2nd cousin through same sex twins with unknown zygosity	$h^2\left[p_{mz}\left(\frac{1+m}{2}\right)^4 + p_{dz}\left(\frac{1+m}{2}\right)^5\right]$
2nd cousin	$h^2\left(\frac{1+m}{2}\right)^5$
Step sibling	$h^2 m\left(\frac{1+m}{2}\right)^2$
Co-cousin	$h^2 m\left(\frac{1+m}{2}\right)^4$

Note. Relation is the category of relative; Predicted correlation is the expression for predicted genotypic correlation in the Fisherian framework. For categories involving twins of unknown zygosity, p_{mz} and p_{dz} are the assumed proportions of same-sex twin pairs who are monozygotic and dizygotic, respectively.

Table S10. Means and standard deviations of standardized scores on 8th grade tests for subsets of students identified as having relatives of a certain category

Relation	Mean	SD	N	%
MZ twin	0.147	0.949	1,597	0.22
UZ twin	-0.111	1.012	11,448	1.59
DZ twin	0.041	0.989	10,382	1.44
Sibling	0.025	0.999	587,835	81.64
Half sibling	-0.173	0.979	216,595	30.08
1st Cousin t/ UZ twins	0.032	0.963	12,098	1.68
1st Cousin	0.051	0.983	614,078	85.29
Half 1st cousin	-0.136	0.975	128,707	17.88
2nd cousin t/ UZ twins	-0.043	0.955	9,863	1.37
2nd cousin	0.003	0.972	418,154	58.08
Step sibling	-0.281	0.948	22,182	3.08
Co-cousin	0.069	0.978	435,846	60.54
Adopted sibling	-0.110	1.037	6,272	0.87
Adopted 1st cousin	0.114	1.001	25,658	3.56
Adopted 2nd cousin	0.000	0.979	21,001	2.92
Adopted co-cousin	0.038	0.995	19,139	2.66

Note. *"Mean" and "SD" are the means and standard deviations of test scores (standardized within the total sample of students) for students identified as having at least one relative of the category specified by the "Relation" column. "N" gives the total number of such students for each category, and "%" gives how large a percentage this number is out of the total sample of students with scores on 8th grade tests, which is 719,984 students.*

SI References

1. S. H. Hurlbert, Pseudoreplication and the design of ecological field experiments. *Ecological monographs, 54*(2), 187-211 (1984).
2. M. Hällsten, Inequality across three and four generations in egalitarian Sweden: 1st and 2nd cousin correlations in socio-economic outcomes. *Research in Social Stratification and Mobility*, *35*, 19-33 (2014).
3. G. Solon, M. E. Page, & G. J. Duncan, Correlations between neighboring children in their subsequent educational attainment. *Review of Economics and Statistics, 82*(3), 383-392 (2000).
